# Discovery of Lactomodulin, a Unique Microbiome-Derived Peptide That Exhibits Dual Anti-Inflammatory and Antimicrobial Activity against Multidrug-Resistant Pathogens

**DOI:** 10.3390/ijms24086901

**Published:** 2023-04-07

**Authors:** Walaa K. Mousa, Rose Ghemrawi, Tareq Abu-Izneid, Azza Ramadan, Farah Al-Marzooq

**Affiliations:** 1 College of Pharmacy, Al Ain University, Abu Dhabi P.O. Box 112612, United Arab Emirates; 2 AAU Health and Biomedical Research Center, Al Ain University, Abu Dhabi P.O. Box 112612, United Arab Emirates; 3 College of Pharmacy, Mansoura University, Mansoura 35516, Egypt; 4 Department of Medical Microbiology and Immunology, College of Medicine and Health Sciences, UAE University, Al Ain P.O. Box 15551, United Arab Emirates

**Keywords:** microbiome, *Lactobacillus*, immunomodulatory, anti-inflammatory, antibiotics

## Abstract

The human body is a superorganism that harbors trillions of microbes, most of which inhabit the gut. To colonize our bodies, these microbes have evolved strategies to regulate the immune system and maintain intestinal immune homeostasis by secreting chemical mediators. There is much interest in deciphering these chemicals and furthering their development as novel therapeutics. In this work, we present a combined experimental and computational approach to identifying functional immunomodulatory molecules from the gut microbiome. Based on this approach, we report the discovery of lactomodulin, a unique peptide from *Lactobacillus rhamnosus* that exhibits dual anti-inflammatory and antibiotic activities and minimal cytotoxicity in human cell lines. Lactomodulin reduces several secreted proinflammatory cytokines, including IL-8, IL-6, IL-1β, and TNF-α. As an antibiotic, lactomodulin is effective against a range of human pathogens, and is most potent against antibiotic-resistant strains such as methicillin-resistant *Staphylococcus aureus* (MRSA) and vancomycin-resistant *Enterococcus faecium* (VRE). The multifunctional activity of lactomodulin affirms that the microbiome encodes evolved functional molecules with promising therapeutic potential.

## 1. Introduction

**Figure 1:**
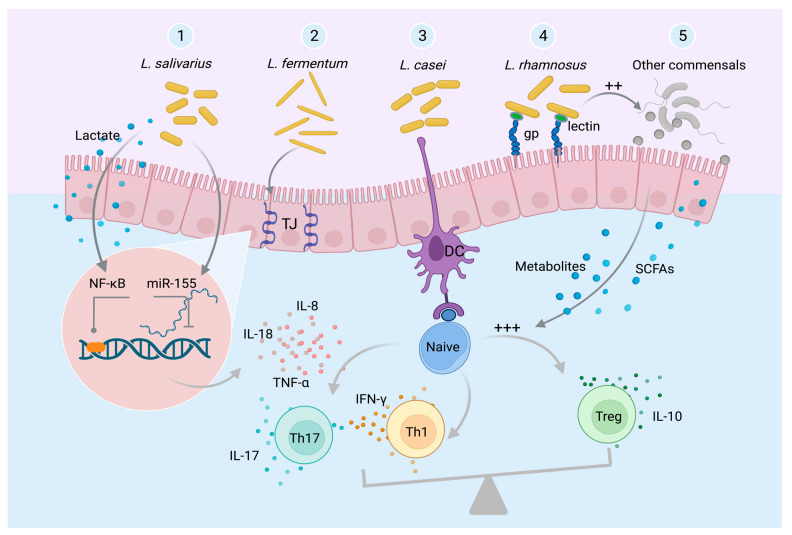
Graphical illustration to summarize some of the mechanisms employed by *Lactobacillus* species to modulate the immune system. (1) Regulation of miRNAs that affect cytokine gene expression and production of lactic acid, which affects the transcriptional regulator NF-κB, where both effects downregulate the production of proinflammatory mediators such as IL8 and TNF-α. (2) Upregulation of the tight junction’s (TJ) expression enhances the gut barrier and prevents leakage of metabolites that can cause inflammation. (3) Enhancement of the differentiation of naïve T cells into Treg cells, subsequently leading to higher production of the anti-inflammatory cytokine IL-10. (4) Expression of lectin-GR-1, which is a surface protein that binds to glycoprotein (gp), increasing adherence to the mucosa and leading to better protection and less inflammation. (5) Enhancement of other commensal microbes, such as those that produce anti-inflammatory molecules or short-chain fatty acids (SCFAs).

The human body is colonized by trillions of microbial species collectively known as the human microbiota [1]. Gut microbes exceed 95% of the entire microbiota, and are considered the most diverse microbial ecosystem on earth [2,3]. The proximity of the commensal gut microbes to the local immune cells in the gut enabled them to undergo co-evolution [4,5], leading to the development of multiple strategies to regulate the immune system [6,7,8,9,10]. These strategies involve the modulation of gut permeability [11], the secretion of specialized molecules [12], and the control of host gene expression [13]. For example, *Lactobacillus* species modulate the immune system through the production of lactic acid, which suppresses the expression of NF-κB, TNF-α, and other cytokines [14,15]. *L. salivarius* and *L. fermentum* modulate the expression of miRNA-155 and 233 to increase the integrity of mucosa, thus preventing leaky gut [16]. *L. casei* maintains TH17/Treg balance, leading to a reduction in proinflammatory cytokines such as IL-6 [17]. *L. rhamnosus* produces Lectin-GR-1, a surface protein that upregulates anti-inflammatory cytokines while simultaneously mediating proper adhesion of Lactobacilli to the epithelial cells, which block pathogen entry and colonization [18]. In addition, *L. rhamnosus* upregulates the genes responsible for mucin production, and thus improves intestinal barrier function, preventing leaky gut and decreasing inflammation [19]. *L. acidophilus* regulates TH17/Treg balance, leading to the suppression of proinflammatory mediators, such as IL-12, IL-6, IL-17, and IL-22, and the induction of the anti-inflammatory cytokine IL-10 [20,21]. In addition, *Lactobacillus* species also promote the growth of other immunomodulatory microbes, including *Rosburia* and *Ruminococcus* [22]. In addition to their immunomodulatory role, many species of Lactobacilli produce a wide range of antimicrobial metabolites, such as acids, hydrogen peroxides, and antimicrobial peptides, as previously reviewed [23]. Figure 1 illustrates some of the immunomodulatory mechanisms of *Lactobacillus* species.

The increase in data linking *Lactobacillus* depletion to the onset and progression of several inflammatory diseases has fueled interest in their potential use as probiotics to supply missing functions and restore a healthy balance to microbial communities. However, there are possible adverse reactions to the use of probiotics, including interactions with existing microbes, such as via horizontal gene transfer [24]. Probiotics can also result in unexpected interactions with the host. For example, *E. coli* Nissle 1917, one of the first probiotics, encodes a gene cluster for the synthesis of colibactin, which has been linked to colorectal cancer [25]. Moreover, probiotics might fail to establish long-term colonization due to competition with indigenous bacteria [26]. Another promising approach involves fecal transplantation to control infectious and inflammatory conditions, such as pseudomembranous colitis, which results from recurrent infection with *C. difficile*. However, this approach is not without challenges, especially regarding safety concerns related to retracting antibiotic-resistant bugs and the unexpected long-term impacts [27]. Alternatively, identifying the chemical mediators of microbiome function is an appealing strategy that could impact the development of microbiome-based therapeutics and could be fully evaluated, tested, and optimized. 

The aim of this study was to identify functional secreted molecules from the gut microbiota that have the potential for development as drugs for the treatment of inflammatory and/or infectious diseases. This study also aimed to develop a strategy to identify these chemical mediators based on computational and experimental protocols. We report the discovery of lactomodulin, a unique peptide produced by the *L. casei* subspecies *rhamnosus.* Lactomodulin exhibits a potent anti-inflammatory effect in suppressing proinflammatory mediators, including IL-8, IL-6, IL-1B, and TNF-α in the Caco-2 cell line, with minimal cytotoxicity. Further investigation of lactomodulin reveals its antimicrobial activity against several human pathogens, including the antibiotic-resistant strains methicillin-resistant *Staphylococcus aureus* (MRSA) and vancomycin-resistant *Enterococcus faecium* (VRE). Our findings highlight the microbiome’s potential in the advancement of drug discovery and development.

## 2. Results

### 2.1. Immunomodulatory Potential of Live Lactobacillus Strains

**Figure 2:**
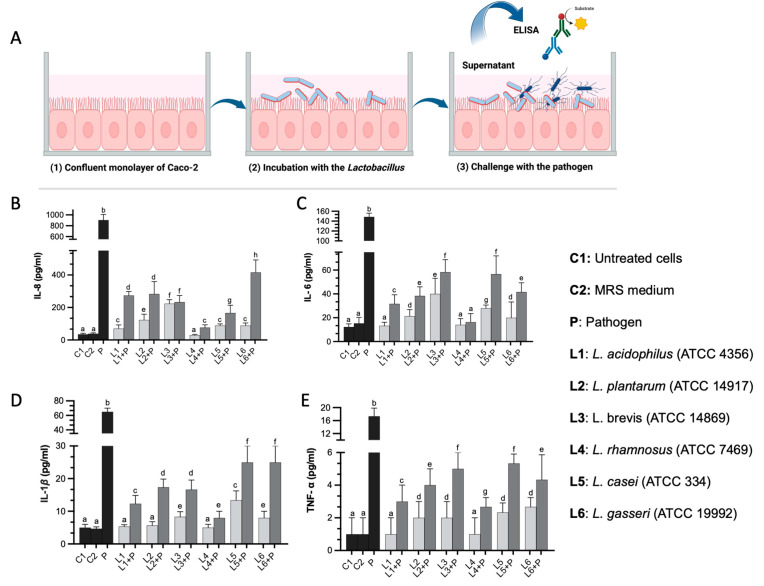
Immunomodulatory activity of 6 *Lactobacillus* strains from different species in a co-culture experimental model using Caco-2 cell lines. (**A**). Graphical illustration demonstrating the co-culture experimental design, which consists of three steps: (1) obtaining a confluent monolayer of Caco-2 cells, (2) incubation with each *Lactobacillus* strain, and (3) challenge with the pathogen to mimic the natural protective mechanism. (**B**–**E**). Graphs show the concentration of secreted cytokines in the cell supernatant with each treatment, as measured using ELISA kits for IL-8, IL-6, IL-1β, and TNF-α, respectively. In each graph, black bars represent the controls, where C1 represents untreated cells, C2 represents MRS medium used in bacterial culture, P represents the pathogen only, light gray bars represent inoculation with each strain without a further challenge with the pathogen (strains are labeled L1 to L6), and dark gray bars represent the co-incubation of each strain and the pathogen. Each measurement was performed in triplicate, and results are shown as mean ± SD. Different letters indicate significant differences at *p* < 0.05.

We selected six *Lactobacillus* strains (L1–L6) for an in-depth investigation of their immunomodulatory activities based on previous disease-association studies [28]. To assess the activity of the selected strains, we designed a co-culture experiment in which we incubated each strain with colon carcinoma cells, Caco-2, followed by a challenge with the pathogen *C. difficile*, which represented gut pathogens known to induce strong inflammatory conditions in the colon. The Caco-2 cell line, either alone or in a co-culture with other cells, is widely used in the screening of immunomodulatory drugs, and some reports suggest that this cell model might be predictive of in vivo behavior [29,30,31]. We conducted the initial screening of live probiotics using a pre-treatment design in which the live strains were incubated first, followed by the pathogen challenge; this design mimics the natural ecological setting in a balanced microbiome in which the beneficial strains are already established before pathogen colonization. Thereafter, we used the cells’ supernatant to measure the secreted amount of IL-8, IL-6, IL-1β, TNF-α, and IL-10 via ELISA (Figure 2A).

Infection with *C. difficile* alone (P) resulted in a significant increase in proinflammatory cytokines, although we were not able to detect IL-10. The most induced cytokine was IL-8, with levels 24-fold those of the untreated cells and the media-only control (C1 and C2, respectively) (Figure 2B). This finding is consistent with previously published data that show that *C. difficile* induces inflammation via potent upregulation of IL-8, IL-1β, and IL-6 [32]. Inoculation with five *Lactobacillus* strains resulted in a one- to five-fold increase in IL-8 compared to the untreated cells, except for L4. These data are consistent with previous reports showing that some *Lactobacillus* strains can result in initial stimulation of the immune response through the induction of IL-8 and IL-1β secretion and the generation of reactive oxygen species [33,34]. Cells incubated with L1-L8 strains prior to the pathogen challenge showed a 50–90% drop in IL-8 secretion. The secreted amounts of the other tested cytokines followed a similar pattern to IL-8. IL-6 secretion increased 11-fold following the pathogen challenge, and dropped to 20%, 25%, 40%, 0%, 40%, and 30% of its augmented value with L1-L6, respectively. IL-1β secretion increased 12-fold with the pathogen, decreased by 60% with L5 and L6, and decreased by 73% with L2 and L3. L1 and L4 resulted in the most significant reduction in IL-1β of 87% (Figure 2D). TNF-α secretion showed a 12-fold increase following the pathogen-only challenge, no difference with L1, and a one-fold increase with the L2-L6 strains (Figure 2E). With *Lactobacillus* treatments, the secreted amount of TNF-α dropped to 70–84%, with the most intense reduction observed with L4 (Figure 2E). Taken together, our data show that all the tested strains resulted in a significant decrease in the measured cytokines following the pathogen challenge; therefore, it is worth further investigating this activity’s mechanism of action to identify whether it occurs due to secreted molecules or whether it is attributed to simple competition between the live strains and *C. difficile*. Previous reports show that some *Lactobacillus* strains reduce the adhesion rate of *C. difficile* and hinder its efficient colonization [35]. Our data also support the idea that not all bacteria can reduce inflammation. For example, *E. coli* and *Enterococcus faecalis* increased the inflammation caused by *C. difficile* when tested using the same experimental design (data are not shown), suggesting that the observed anti-inflammatory activity of the tested strains might be a unique feature. 

### 2.2. Combined Experimental/Computational Approach to Identifying the Functional Chemical Mediators

In our co-culture experiment, the L4 strain showed the most significant activity in suppressing the release of all measured proinflammatory cytokines; this neutralized the inflammatory effect of the pathogen at the basal level of untreated cells for IL-8, IL-6, and IL-1β, and resulted in the most significant decrease in TNF-α occurring in L4 compared to all the other strains. Moreover, incubation with L4 did not result in an initial increase in the measured proinflammatory cytokines, in contrast to the other strains, which showed at least a one-fold increase in at least one cytokine secretion compared to the untreated control cells or media only. For these reasons, we selected L4 for further investigation of the chemistry behind this activity. First, to verify whether the observed activity was mediated by a secreted metabolite, we tested the activity of the cell-free extracts using the same experimental design, followed by measuring the amount of secreted IL-8. The data showed that the cell-free extract of L4 reduced IL-8 secretion by 84% of its induced level with *C. difficile*. Notably, the cell-free extracts of all the other strains were not shown to significantly reduce IL-8 compared to the corresponding live strains.

**Figure 3:**
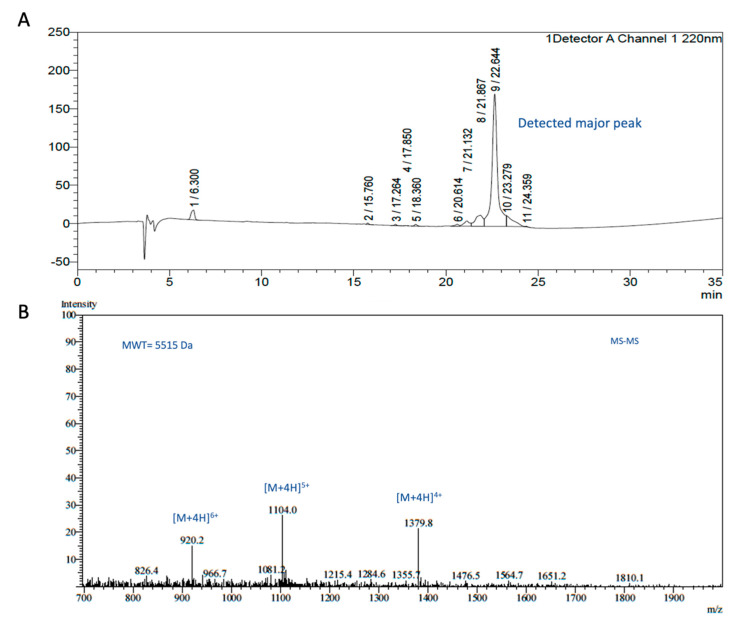
Identification of a potential molecule from L4 driving its anti-inflammatory potential. (**A**,**B**) HPLC and MS-MS spectra of the peptide, respectively.

For a more in-depth investigation, we scaled up the culturing of L4 (*L. rhamnosus*). We cultured *L. rhamnosus* in 3 L of MRS medium for 24 h at 37 °C, and collected the metabolites in a mixture of HP20, XAD7, and XAD16 resins. The crude extract was fractionated on a reverse phase C18 column. The generated fractions were tested for immunomodulatory activity using the same experimental procedure, and IL-8 was measured as an indicator of activity. Out of the twelve fractions, three showed a significant reduction in IL-8 production, with one fraction resulting in 100% neutralization of IL-8 by the pathogen alone. Further LC/MS-MS examination of this fraction revealed a major compound with an MW of 5515 Da and a fragmentation pattern corresponding to the peptide compound (Figure 3). To predict the sequence of the peptide, we analyzed the genome of L4 (*Lactobacillus casei* subsp. *rhamnosus*, ATCC 7469) using DeepRipp software [36], a deep learning tool that we previously developed to predict unmodified and post-translationally modified peptides from genomic or metagenomic data. DeepRipp generates a confidence score and structure prediction of the encoded peptides and links the computational prediction from the genome to the possible corresponding mass in LC/MS-MS data. Our investigation led to the identification of the sequence of an unmodified peptide with a theoretical MW of 5516.32 Da, matching the observed MW for the candidate active molecule in the extract. The sequence for this encoded peptide is MNKLNEVELSKISGGIGPLVMNKLNEVELSKISGGIGPLVIPVAAILGFLATDAWNHADELVAGVKQGWERS. Our investigation of the genome and cell-free extracts of the other strains did not show evidence of the presence of this unique metabolite. To further investigate the biological activity of the identified peptide, we synthesized it through Genscript and named it lactomodulin. 

### 2.3. Lactomodulin Is a Unique Multifunctional Molecule with Minimal Toxicity in Cell Lines

**Figure 4:**
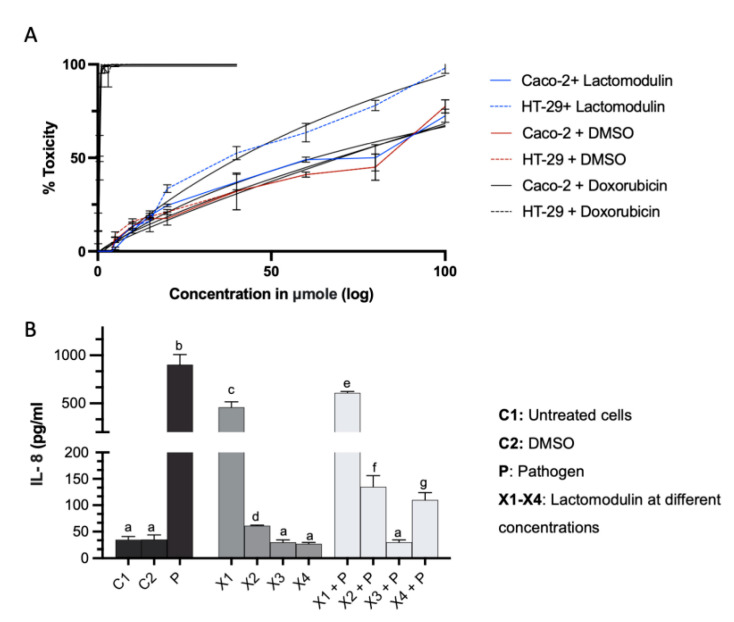
Minimal cytotoxicity of lactomodulin against two colon cell lines and reduction in IL-8 secretion. (**A**) determination of IC_50_ of lactomodulin in HT-29 and Caco-2 cell lines. (**B**) Determination of the most effective dose of lactomodulin in reducing IL-8 secretion, where X1–X4 represent lactomodulin at different concentrations of 50, 10, 1, and 0.5 μmol, respectively. Each measurement was performed in triplicate, and results are shown as mean ± SD. Different letters indicate significant differences at *p* < 0.05.

Before evaluating the anti-inflammatory activity of lactomodulin, we first tested the cytotoxicity of the peptide on two different GIT-derived cell lines (Caco-2 and HT-29) using an MTT assay. The IC_50_ of lactomodulin against Caco-2 was 60 μmol, compared to 40 μmol for HT-29 (Figure 4A), indicating that lactomodulin toxicity is minimal. Our data suggest that lactomodulin exerts higher toxicity in HT-29, which is consistent with a previous suggestion of elevated sensitivity in HT-29 to toxins compared to Caco-2 [37]. To further investigate the anti-inflammatory activity of the pure molecule, we first tested the ability of four different concentrations of lactomodulin below the IC_50_ value in Caco-2 (50, 10, 1, and 0.5 μmol) to decrease IL-8 secretion using the pre-treatment experimental model. We experimentally determined the tested concentration using a panel of serial dilutions and the range of effective concentrations that matched the live strain inhibition level. The results showed that lactomodulin at a 50 μmol concentration increased inflammation up to 12-fold compared to the untreated cells, while 10 μmol resulted in a 0.74-fold increase. Neither the 1 nor the 0.5 μmol concentration of lactomodulin showed a significant difference compared to untreated cells (Figure 4B). When challenged with the pathogen, all concentrations resulted in a significant drop in IL-8 secretion. The 50 μmol concentration showed the lowest level of activity, decreasing IL-8 to 32%, followed by concentrations of both 10 and 0.5 μmol, which decreased IL-8 to ~85%. The 1 μmol concentration of lactomodulin neutralized the IL-8 level to a baseline of untreated cells without any initial increase in inflammation and was thus selected for further experiments (Figure 4B).

**Figure 5:**
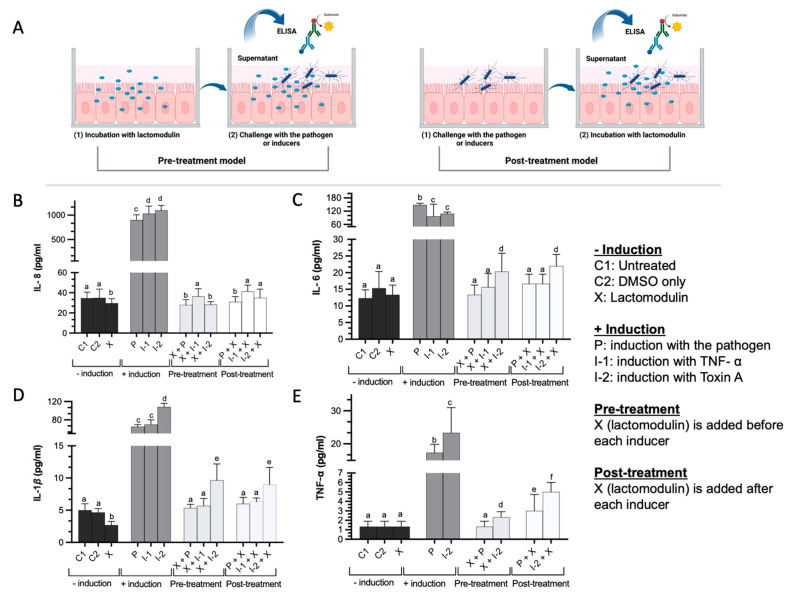
Potent anti-inflammatory activity of lactomodulin in Caco-2 cell line model system. (**A**) Graphical illustration shows the two different experimental designs: the pre-treatment model in which the cells are first treated with the compound and then the pathogen, and the post-treatment model in which the cells are first challenged with the pathogen or inducers and then treated with the compound. (**B**–**E**) Graphs showing the effect of lactomodulin on the 4 measured cytokines. Each graph shows 4 groups of data: (1) black bars: cells without inducers; (2) dark gray bars: cells challenged with the pathogen (P), TNF-α (I-1, 20 ng/mL), and Toxin A (I-2, 10 ng/mL); (3) light gray bars: pre-treatment; and (4) whitish bars: post-treatment data. Each measurement was performed in triplicate, and results are shown as mean ± SD. Different letters indicate significant differences at *p* < 0.05.

For a more in-depth understanding of the anti-inflammatory activity of lactomodulin, we tested the compound using two different models: (1) a pre-treatment model in which 1 μmol of lactomodulin was added 6 h before stimulation with different inducers, followed by incubation for a total of 18 h, and (2) a post-treatment model in which the cells were first stimulated for 2 h with inducers, followed by the addition of lactomodulin at the same concentration and a total incubation time of 18 h (Figure 5A). To rule out an indirect effect of lactomodulin on the pathogen, we used three different inducers of inflammation: (1) *C. difficile* (P), (2) TNF-α (I-1), and (3) purified toxin A from *C. difficile* (I-2). Thereafter, we measured the amount of secreted IL-8, IL-6, IL-1β, and TNF-α using ELISA. Overall, lactomodulin inhibited or significantly decreased all the tested cytokines in both models and with all the inducers used. The induced cells showed a 24- to 27-fold increase in IL-8 production with different inducers (Figure 5B). Both pre-treatment and post-treatment with lactomodulin neutralized IL-8 to a baseline of untreated cells. We observed a similar trend with IL-6, IL-1β, and TNF-α, and noted that lactomodulin is more effective at neutralizing the effect of the pathogen or TNF-α compared to toxin A, with a minor (~6–7% less activity) but statistically different value of *p* < 0.05 (Figure 5C–E). For example, the reduction in IL-1β secretion with lactomodulin was 94% in both models when the cells were challenged with toxin A, compared to 100% when the cells were induced with the pathogen or TNF-α. We did not detect IL-10 in the supernatant in cells treated with lactomodulin alone or with the inducers. Although we did not detect an increase in IL-10 upon treatment with the live strains or the pure peptide, a previous study claimed strong anti-inflammatory activity of a protein fraction from *L. plantarum* in murine cells [38].

**Figure 6:**
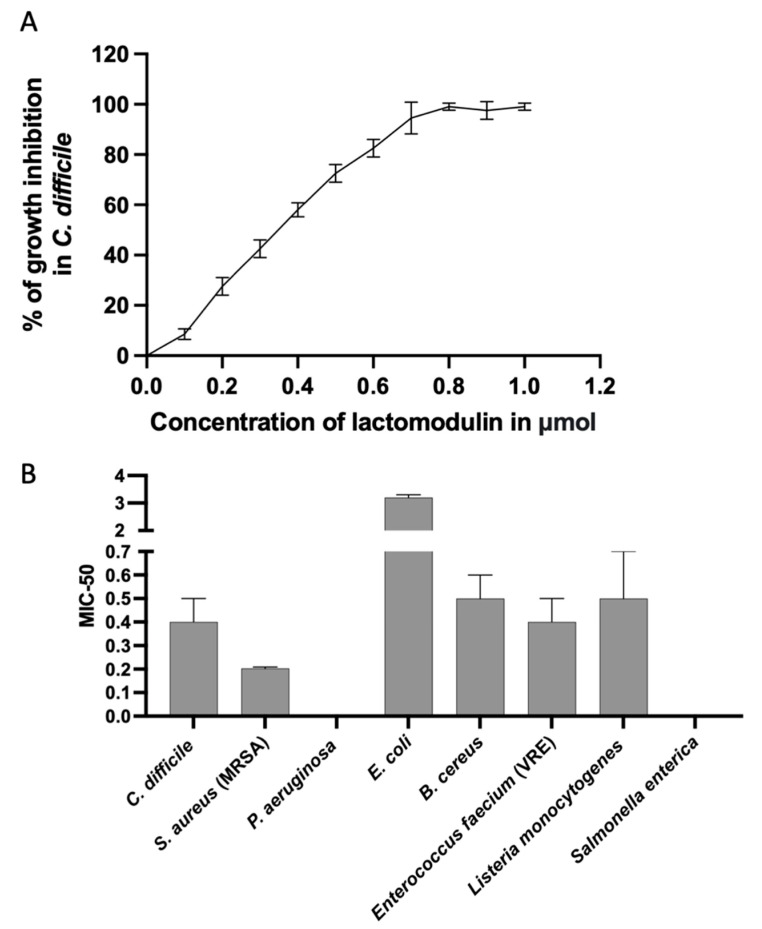
Lactomodulin exerts antibiotic-like action against a broad range of human pathogens. (**A**) MIC_50_ of lactomodulin in *C. difficile* with % of growth inhibition at different concentrations. (**B**) The activity spectrum of lactomodulin in some human pathogens with broad activity against Gram-positive bacteria, including antibiotic-resistant strains. All data shown are averages of 3 replicated independent experiments.

Given the nature of lactomodulin and how it compares to similar peptides, we tested whether the compound exerts antibiotic-like activity against *C. difficile*. We tested the inhibitory activity of the compound against *C. difficile* using the agar diffusion method, in which it showed strong antibacterial activity (Figure 6A). We then conducted a broth microdilution assay to determine the minimal inhibitory concentration (MIC). The data showed that MIC_50_ of lactomodulin was 0.4 μmol (Figure 6B), which is comparable to previously characterized antimicrobial peptides [39]. To understand the mode of action of lactomodulin, we determined its minimal bactericidal concentration in *C. difficile*. We observed no growth on agar plates with 0.9 μmol of lactomodulin, suggesting that it has a bactericidal nature. To identify the spectrum of activity, we tested the compound on different human pathogens, including methicillin-resistant *Staphylococcus aureus* (MRSA), *Pseudomonas aeruginosa*, *E. coli*, vancomycin-resistant *Enterococcus faecium* (VRE), *Listeria monocytogenes*, and *Salmonella enterica*. The results suggest that lactomodulin exerted potent antibiotic activity, mostly against Gram-positive bacteria, and was highly effective against the antibiotic-resistant strain (MRSA) (Figure 6C). The highest MIC_50_ of lactomodulin was observed in MRSA with a value equal to 0.2 μmol, followed by *C. difficile* and VRE, with values of 0.4 μmol, and then *B. cereus* and *L. monocytogenes* at 0.5 μmol. Lactomodulin did not show activity against Gram-negative bacteria, including *P. aeruginosa* and *S. enterica,* but it showed activity against *E. coli* at a high concentration, 14 times its MIC_50_ in MRSA. The positive control antibiotics used in this study were metronidazole, daptomycin, amoxycillin, and ciprofloxacin at concentrations of 1–5 μg/mL. We tested lactomodulin for inhibitory activity against fungi, including *Candida albicans* and *Saccharomyces cerevisiae,* but it lacked antifungal activity, even at high concentrations of up to 20 times its average MIC_50_, against Gram-positive bacteria.

## 3. Discussion

The microbiome encodes millions of cryptic small molecules that have evolved to recognize specific cellular targets and mediate health or disease [40,41]. Mining these microbiome-derived molecules will lead to the discovery of unique therapeutics and diagnostic biomarkers [42,43]. Here, we report the discovery of lactomodulin, a new microbiome-derived molecule with dual activity as a potent anti-inflammatory and antimicrobial agent that exhibits minimal cytotoxicity in contrast to conventional antibiotics. Given this unique functionality, we hypothesize that lactomodulin has an evolutionary role in enhancing the ecological fitness of the producer and enabling host colonization. Suppressing host inflammation is a prerequisite to creating tolerance and maintaining long-term symbiosis, while antibiotic function enhances competitiveness and allows the producer to exert control over the microbial population in the gut and/or to kill invading pathogens. Several examples have been reported that highlight these strategies in microbe–host interactions [44].

### 3.1. Lactomodulin Is a Promising Drug Lead for Further Development

Our investigation of lactomodulin activity shows that it can suppress proinflammatory cytokines regardless of the etiology of inflammation. In our experimental design, we induced inflammation using co-infection with a pathogen or its purified toxin, in addition to TNF-α, which is known to stimulate a wide inflammatory cascade through activation of the NF-κB signaling pathway [45]. The level of TNF-α in the body increases in response to various inflammatory triggers, such as autoimmune diseases, trauma, and infection/sepsis [46]. These findings suggest that lactomodulin could have wide application in a variety of inflammatory conditions, such as inflammatory bowel diseases, autoimmune conditions, gastritis, pseudomembranous colitis, and other infection-induced inflammation [47]. The antibiotic activity of lactomodulin against resistant strains, such as MRSA and VRE, is interesting, given that the current antibiotic resistance crisis is projected to kill 10 million by 2050, with the depletion of effective antibiotics and an increase in multidrug-resistant bacteria. MRSA and VRE are classified by the World Health Organization as priority pathogens [48]. MRSA is a public health threat; with the recent expansion in infection rates and the increased burden on healthcare, at least 80,000 infections have occurred in the US alone, and mortality rates are escalating [49]. Its dual functionality, combined with its lack of intense cytotoxicity, makes lactomodulin interesting for further development. We plan to further investigate lactomodulin, including studying its mechanism of action, developing a computational design of its shorter fragments, carrying out testing for maintaining/enhancing its activity or its cytotoxicity profile, and studying the probability of developing resistance.

### 3.2. Microbiome-Derived Peptides Are Often Multifunctional with a Unique Mode of Action

The few examples of functional peptides identified from the human microbiota show their unique functionality and their apparent evolved role in the recognition of specific cellular targets or combating host pathogens. For example, lugdunin is produced from commensal *Staphylococcus lugdunensis* in the nose and kills *Staphylococcus aureus* with a low probability of developing cross resistance due to the high selectivity of the discovered self-resistance mechanism [50]. In addition to its antibiotic activity, lugdunin increases immune stimulation by upregulating IL-37 and CXCL8/MIP-2 in human keratinocytes and recruiting monocytes and neutrophils in vivo [51]. Lugdunin exerts its antibacterial action without forming pores in the cell membranes, in contrast to the common mode of action of antimicrobial peptides. Specifically, it breaks down the electrical potential of the cell membrane through the translocation of protons across the membrane [52]. However, it is still not known whether lugdunin itself acts as an ion transporter. Another example of microbiome-derived peptides includes the identification of two peptides (HG2 and HG4) with potent anti-inflammatory and antimicrobial activity against MRSA infection in vitro and in a *Galleria mellonella* model [53]. HG2 and HG4 exhibited their activity by selectively binding to bacterial lipids, thus affecting membrane permeability and, in parallel, decreasing the concentration of ATP inside the cell [53]. However, this action is selective toward bacterial lipids over human lipids. Another proline-rich antimicrobial peptide derived from insects, named Api137, exerts antimicrobial activity by trapping the ribosomal release factors RF1 or RF2 in *E. coli*, which prevents ribosomal termination and, consequently, inhibits protein synthesis [54]. Following a computational drug design approach, a group of researchers developed antimicrobial peptides with broad activity against multidrug-resistant pathogens. Interestingly, these peptides not only disrupted cell membranes, but also modulated microbial gene expression by upregulating genes, such as those related to stress response proteins, virulence, or membrane integrity, while downregulating others, such as genes that encode transporter proteins [55].

### 3.3. Microbiome Peptides Are a Promising Approach to Drug Development

Microbial peptides are a promising avenue for drug discovery and development because of their highly specific cellular targets, complex mode of action, multi-functionality, and pharmacodynamic properties, which decrease the rate of resistance against them [56,57,58,59] compared to conventional antibiotics. Additionally, microbial peptides have the added advantage of exhibiting a synergistic effect when combined with other antibiotics. There is interest in developing microbiome-derived peptides as antibiotics because of their varied and complex mode of action, which decreases their rate of resistance [60,61]. However, the applications of these peptides extend beyond antibiotics to include many functionalities, such as antiviral, antifungal, and anticancer agents [62]. This wave of renewed interest is accompanied by the accelerated development of computational tools to compute the discovery of these peptides from microbial genomes [63,64,65,66], and to further optimize their formulation to decrease toxicity and increase their stability as drug leads [67] through chemical modification, novel designs, and new delivery systems [55,56,57,58,59,60,61,62,63,64,65,66,67,68]. Other studies have developed mathematical models to predict the antimicrobial activity and structure–activity relationships of peptides from their tertiary structures [69]. A study computationally identified 2349 putative peptides and synthesized 216 individual sequences, and reported that 83% of these peptides exhibit antimicrobial activity, including against antibiotic-resistant pathogens [70]. Identifying the secreted chemicals that mediate the diverse functions of the microbiome is an appealing strategy that could impact the development of microbiome-based therapeutics and could be fully evaluated, tested, and optimized.

## 4. Materials and Methods 

### 4.1. Microorganisms and Culture Conditions

We used 6 *Lactobacillus* strains in this study based on their beneficial effects, as reported in previous studies [71]. These strains were *L. acidophilus* (ATCC 4356), *L. plantarum* (ATCC 14917), *L. brevis* (ATCC 14869), *L. casei,* subspecies *rhamnosus* (ATCC 7469), *L. casei* (ATCC 334), and *L. gasseri* (ATCC 19992). *Lactobacillus* strains were purchased from Microbiologics, St. Cloud, MN, USA. All strains were grown in de Man, Rogosa, and Sharpe broth (MRS media) for 24 h at 37 °C under anaerobic conditions. To obtain a cell-free supernatant, the culture was centrifuged at 16,000 rpm for 5 min, followed by filtration using a sterile bacterial filter (Merck Millipore, Darmstadt, Germany). We cultured *C. difficile* (ATCC 43602) in meat extract media under anaerobic conditions, while fungi, including *C. albicans* (ATCC18804) and *Saccharomyces cerevisiae* (ATCC 18824), were grown in Sabouraud dextrose broth under aerobic conditions. The pathogens used in this study, in addition to *C. difficile*, included methicillin-resistant *Staphylococcus aureus* (MRSA, BAA-2313), *Pseudomonas aeruginosa (BAA-1744)*, *E. coli* (ATCC 33694), vancomycin-resistant *Enterococcus faecalis* (VRE, BAA-2317), *Listeria monocytogenes (ATCC 15313)*, and *Salmonella enterica* (ATCC 13314). Each pathogen was cultured in the recommended medium. For long-term storage, an aliquot of each culture was maintained in 50% glycerol and stored at −80 °C.

### 4.2. Cell Lines and Culture Conditions

The cell lines used in this study were human colorectal adenocarcinoma Caco-2 (cancer coli-2) and colorectal adenocarcinoma cells HT-29. All cell lines were cultured in Dulbecco’s modified Eagle’s medium (DMEM) supplemented with the following: (1) 10% fetal bovine serum, (2) antibiotics (100 U/mL penicillin and 100 mL/mL streptomycin), and (3) 25 μg/mL amphotericin B at 37 °C in an atmosphere of 5% CO_2_ (Eppendorf, Hamburg, Germany). Cells were incubated inside a 5% CO_2_ incubator at 37 °C for 5 days until they reached 90% confluence. Thereafter, we used PBS buffer to wash cells 2–3 times to get rid of the culture media or non-adherent cells. Before each assay, cells were seeded in 48-well tissue-culture plates (BD Falcon, Becton, NJ, USA) and incubated for 24 h inside a 5% CO_2_ incubator at 37 °C (at a density of 50,000 cells per well). 

### 4.3. Large-Scale Culturing and Metabolite Extraction of L. rhamnosus

To test whether the strain that exhibited the most anti-inflammatory activity mediated this action through secreted molecules, we tested the activity of the bacterial extract using the same experimental design. Three liters of the *L. rhamnosus* strain was grown in MRS media for 48 h without shaking; then, the produced metabolites were collected in a mixture of synthetic resin, including HP20, XAD7, and XAD16 (Sigma-Aldrich, St. Louis, MO, USA), with 60 g of resin per liter of the culture, and shaken for 2 h. Resin was collected via filtration, washed twice with water, and then eluted with methanol. The methanol extract was then concentrated to dryness, and the residue was redissolved in water/acetonitrile and fractionated using a Biotage automated chromatography system on reversed phase C18 silica using water/acetonitrile as a mobile phase (20:100%); this generated 10 fractions. In parallel, we created two other preparations via precipitation of the cell-free supernatant using ammonium sulfate to enrich the proteins. All 12 fractions were then tested for immunomodulatory activity.

### 4.4. Computational Prediction of the Active Metabolite

The analysis of the most active fraction using LC/MS-MS revealed a major peak with an MWT of 5515 Da and MS-MS data indicative of a peptide compound. To predict the possible sequence of this peptide, we used DeepRipp software (https://openebench.bsc.es/tool/deepripp accessed on 3 January 2022). DeepRipp is a deep learning algorithm that predicts modified and unmodified microbial peptides from genomic or metagenomic sequences. After the genome has been uploaded to the software, the results are displayed as a list of predicted sequences, confidence scores, and similarity scores compared to previously known peptides. Thereafter, the software links the predicted sequences to potential ions in the LC/MS-MS data. Using the whole genome sequence of the *L. rhamnosus* strain, DeepRipp revealed a possible peptide with a sequence of MNKLNEVELSKISGGIGPLVIPVAAILGFLATDAWNHADELVAGVKQGWERS. To confirm the identity of the peptide, we synthesized the compound using Genscript Inc. (Piscataway, NJ, USA), and then performed a detailed biological evaluation of the peptide, including immunomodulatory, antibiotic activity, and cytotoxicity test.

### 4.5. Co-Culture Experimental Design

To test the immunomodulatory effects of the tested strains, we designed a preventive model co-culture experiment. First, for the live strains, we centrifuged the bacterial cultures (all strains used in this study, including the pathogens) at 12,000× *g* for 2 min, washed them twice with PBS buffer, and finally, suspended the pellets in MRS medium for *Lactobacillus* strains and in meat broth for *C. difficile*. We used 1 µL of 0.5 OD_600_ culture for each 300 µL culture medium in each well of the 12-well plate. For the extract fractions, we used 1 µL of 10 μg/µL. We incubated Caco-2 cells with the live Lactobacilli for a total of 18 h. For the pure peptide, we paired this design with another curative model in which incubation with the peptide was allowed after a 2 h challenge with the pathogen, or 1 μL of recombinant TNF-α (20 ng/mL, Sigma-Aldrich, St. Louis, MO, USA) or toxin A (10 ng/mL, Sigma-Aldrich, St. Louis, MO, USA). The experiment was conducted using antibiotic-free medium. The negative control experiments included untreated cells or cells treated with media blanks for the strains, or with DMSO for the fractions, while the positive control comprised cells treated with the inducers only. The choice of the duration of each incubation was based on prior optimization experiments and previous reports on the optimum timing for cytokine release and measurement in Caco-2.

### 4.6. Determination of Cytokines Concentration in the Supernatant of Caco-2 Cells

To determine cytokine release in Caco-2 cells after co-incubation, we used an enzyme-linked immunosorbent assay (ELISA) for IL-6, IL8, IL-1B, IL-10, and TNF-α on the collected supernatant. We used commercial kits from Invitrogen, Carlsbad, CA, USA (catalogue # BMS213HS, BMS204-3, KHC001, BMS215-2, and BMS223-4 for IL-6, IL8, IL-1B, IL-10, and TNFα, respectively). The released cytokines were measured according to the described protocol. We performed each analysis in three independent replicates.

### 4.7. Cytotoxicity Assay

To assess the cytotoxic effects of the *Lactobacillus* strains and the active peptide, we used an MTT (3-[4,5-dimethylthiazol-2-yl]-2,5 diphenyl tetrazolium bromide) assay on Caco-2 and HT-29 cell lines. Cells were grown in Dulbecco’s modified Eagle’s medium (DMEM) supplemented with 10% fetal bovine serum and antibiotics (100 U/mL penicillin and 100 mL/mL streptomycin) in a humidified incubator (5% CO_2_ in air at 37 °C). Each cell line was plated in 96-well plates (30,000 cells/200 μL in each well) and placed in a CO_2_ incubator for 24 h. After removing the supernatant and washing twice with PBS1X, 20 μL of MTT solution was added to the medium and incubated for 3 h. Finally, the absorbance intensity was measured via spectrophotometry using a microplate reader at an absorbance level of 570 nm (Thermo Fisher Scientific, Waltham, MA, USA). We used DMSO as a negative control.

### 4.8. Assessment of Antimicrobial Activity 

To test whether the peptide possessed antibiotic activity against *C. difficile* and other human pathogens, we performed an agar diffusion test. Briefly, 10 μL of culture of each pathogen, grown actively overnight, was plated on the top of agar plates (using different media that support the growth of each pathogen tested); then, holes were punctured in the agar using a sterile glass pipette, and 20 μL of 1–5 μM lactomodulin was applied into the holes. The plates were incubated anaerobically or aerobically, depending on the required growth conditions of each pathogen, at 37 °C for 24 h. Thereafter, the plates were screened for the presence of any zones of inhibition. To determine the minimum inhibitory concentration (MIC_50_) of lactomodulin, we conducted a broth microdilution antimicrobial assay in a 96-well microtiter plate. Briefly, a single colony of each pathogen was grown for 24 h in its respective broth medium, and then diluted with the same medium in a 1:10,000 ratio (following the McFarland Standards). Thereafter, 196 μL of this inoculated medium was added to each well, and 4 μL of the different serial dilutions of lactomodulin were added to the well, resulting in a final concentration range of 100 μM to 100 nM. The blank control wells contained 196 μL non-inoculated medium and 4 μL DMSO (solvent used to solubilize lactomodulin). Fidaxomicin (1 μM), amoxicillin (5 μM), and ciprofloxacin (2 μM) were used as positive controls. The plates were incubated anaerobically at 37 °C. After 24 h, the OD_600_ of each well was measured using a microplate reader. Thereafter, MIC_50_, defined as the lowest concentration of the peptide that results in 50% growth inhibition, was measured for each pathogen. Each concentration was tested in triplicate, and the entire assay was independently duplicated. The percentage of growth inhibition was determined according to the following equation:

% of inhibition= 1 − [(OD_600_ test − OD_600_ blank medium)/(OD_600_ pathogen only − OD600 blank medium)] × 100

To determine the minimal bactericidal concentration of lactomodulin in *C. difficile*, we spotted 10 µL of the inhibitory concentrations from the broth microdilution experiment (in which we observed no growth) on meat agar supplemented with 5% defibrinated sheep blood, incubated anaerobically for 48 h at 37 °C; then, we observed the plates for the presence of any colonies. The controls included a culture treated with Fidaxomicin (1 μM) and an untreated culture. 

### 4.9. Statistical Analysis

To analyze the data and construct graphs, we conducted a one-way ANOVA using GraphPad Prism V9 (GraphPad Software, La Jolla, CA, USA). All experiments were conducted in triplicate, and the data were presented as means ± standard deviation (SD). Results with *p* < 0.05 were considered statistically significant. Different letters indicate significant differences at *p* < 0.05.

## 5. Conclusions

We report the discovery of lactomodulin, a unique functional peptide from the *L. rhamnosus* bacterium. Lactomodulin exhibited a potent anti-inflammatory effect in suppressing the proinflammatory mediators of the Caco-2 cell line, both as a preventive and treatment intervention, with minimal cytotoxicity. Further investigation of lactomodulin revealed its antimicrobial activity against several human pathogens, including methicillin-resistant *Staphylococcus aureus* (MRSA) and vancomycin-resistant *Enterococcus faecium* (VRE). Our findings highlight the microbiome’s potential in advancing drug discovery.

## Data Availability

No other supporting data have been generated in this study.
